# A Revised Structure for the Glycolipid Terminus of *Escherichia coli* K5 Heparosan Capsular Polysaccharide

**DOI:** 10.3390/biom10111516

**Published:** 2020-11-06

**Authors:** Lufeng Yan, Li Fu, Ke Xia, Shiguo Chen, Fuming Zhang, Jonathan S. Dordick, Robert J. Linhardt

**Affiliations:** 1 College of Biosystems Engineering and Food Science, National-Local Joint Engineering Laboratory of Intelligent Food Technology and Equipment, Zhejiang University, Hangzhou 310058, China; 11513022@zju.edu.cn (L.Y.); chenshiguo210@163.com (S.C.); 2 Department of Chemical and Biological Engineering, Center for Biotechnology and Interdisciplinary Studies, Rensselaer Polytechnic Institute, Troy, NY 12180, USA; lfu@smithfield.com (L.F.); xiak@rpi.edu (K.X.); Zhang2@rpi.edu (F.Z.); dordick@rpi.edu (J.S.D.); 3 Department of Chemistry and Chemical Biology, Center for Biotechnology and Interdisciplinary Studies, Rensselaer Polytechnic Institute, Troy, NY 12180, USA

**Keywords:** heparosan capsular polysaccharide, glycolipid terminus, structure revision

## Abstract

The structure of heparosan capsular polysaccharide (CPS) has been determined using enzymatic digestion with nuclear magnetic resonance (NMR) spectroscopy and mass spectrometry. Previous errors in the assignment of the glycolipid acceptor structure, from which heparosan is extended, have been corrected. The structure of heparosan CPS is GlcNAc *α*-1,[4GlcA *β*-1,4GlcNAc *α*-1,]_n_4GlcA *β*-1,[4Kdo *β*-2,7Kdo *β*-2,]_0 or 1_4Kdo *β*-2,7Kdo *β*-2,4Kdo *β*-2,7Kdo *β*-2,4Kdo *β*-2,7Kdo *β*-2,4Kdo *β*-PG-I (C16:0 or C18:0) (where n is ~250 for a CPS of 100 kDa).

## 1. Introduction

A polysaccharide capsule, anchored in their outer cell membrane, surrounds Gram-negative bacteria, such as *Escherichia coli* [1]. These capsular polysaccharides (CPSs) protect pathogenic bacteria against the host immune response by preventing phagocytosis and protecting against the complement system [2,3]. Often, CPS resembles the polysaccharides found in the host and serves as molecular camouflage preventing the host from recognizing bacteria as foreign [4]. Heparosan, the CPS of *Escherichia coli* K5, has the repeating structure →4)-*β*-d-glucuronic acid (GlcA) (1→4)-*α*-*N*-acetyl- d-glucosamine (GlcNAc) (1→ and resembles the first polysaccharide intermediate in the biosynthesis of heparan sulfate, a glycosaminoglycan that performs important biological functions in all animals [5]. A major difference between this heparosan intermediate in animals and the heparosan CPS in bacteria is the acceptors on which they are biosynthesized [4,5]. In animals, the heparosan is assembled one saccharide unit at a time through the action of polysaccharide synthases on an acceptor corresponding to the tetrasaccharide linkage region attached to the serine residue of a core protein to form a nascent proteoglycan that is subsequently fully elaborated to afford the heparan sulfate proteoglycan [6]. The biosynthesis of heparosan CPS in *Escherichia coli* K5 similarly takes place but instead on a glycolipid acceptor. While bacterial biosynthesis of heparosan CPS has been well studied, the exact structure of the glycolipid acceptor has only recently been elucidated [7]. The study clarified that the glycolipid terminus of *Escherichia coli* K5 heparosan is composed of several linearly linked 3-deoxy-d-*manno*-oct-2-ulosonic acid (Kdo) residues with a phosphatidylglycerol (glycerol- phosphate-glycerol) and monoacyl or diacyl fatty acids [7].

In the current study, we have employed sophisticated enzymatic and spectroscopic tools to investigate the structure of heparosan CPS [8]. It is critical to know the exact structure of *Escherichia coli* K5 heparosan, as it is currently used as an intermediate in the chemoenzymatic synthesis of heparan sulfate and the structurally related anticoagulant drug heparin [9,10]. Briefly, base hydrolysis of *N*-acetyl groups and their subsequent *N*-sulfonation are used to prepare *N*-sulfoheparosan from heparosan. By controlling these reactions, hybrid structures, common to heparan sulfate and heparin, can be prepared. These two kinds of glycosaminoglycans primarily differ by their ratio of GlcNAc and *N*-sulfoglucosamine (GlcNS) residues [11], and contain different contents of other frequent modifications, including epimerization of GlcA to iduronic acid (IdoA) and *O*-sulfation of the 2-position of IdoA residues and the 6-position of the GlcNAc or GlcNS residues. In addition to these major modifications, there exist less frequent modifications such as 3-*O*-sulfation of the GlcNS residues and 2-*O*-sulfation of the GlcA residues [12]. During the process, the glycolipid terminus needs to be under-control, since it is not an expected structural domain in the final heparan sulfate and heparin products. Additionally, carbohydrate-containing macromolecules of bacterial cell surfaces are important in host–pathogen interactions [13], and when their structure is known can be successfully used to prepare vaccines targeting pathogens [14].

## 2. Materials and Methods

### 2.1. Materials

*Escherichia coli* K5 was fermented on glucose as previously described [15]. When *Escherichia coli* K5, producing heparosan capsular polysaccharide, was ready to harvest, cell culture was transferred into tubes and centrifuged at 4000× *g* for 30 min. Then the supernatant was dialyzed in an 8 kDa molecular weight cut-off (MWCO) dialysis bag (MilliporeSigma, Burlington, MA, USA) with flowing water for 2 days. The dialyzed sample was finally lyophilized to obtain salt-free heparosan CPS. Heparin lyase III, originating from *Flavobacterium heparinum*, was expressed and purified in *Escherichia coli* in our laboratory as described previously [16]. Phospholipases A1, A2, and Kdo standard were purchased from Sigma Aldrich (St. Louis, MO, USA). Heparosan disaccharide (ΔUA-GlcNAc) and tetrasccharide (ΔUA-GlcNAc-GlcA-GlcNAc) standard were obtained from heparin lyase III digestion of heparosan CPSs. 1 kDa MWCO spin columns were purchased from MilliporeSigma (Burlington, MA, USA). All other chemicals and reagents were of analytical grade and purchased from commercial corporations.

### 2.2. Heparin Lyase III Digestion

Five hundred milligrams of salt-free heparosan CPSs from *Escherichia coli* K5 was dissolved in 25 mL digestion buffer (50 mM ammonium acetate, 2 mM calcium chloride, pH 7.2), and 10 U heparin lyase III (1 U will form approximate 60 uM of unsaturated uronic acid per hour) was added to get a full digestion at 37 °C overnight until there were no obvious oligomers like tetrasccharides (the evaluation of the tetrasaccharides and longer oligosaccharides was based on a partial digestion of heparosan as shown in Figure S1).

### 2.3. HPGPC Profiles

HPGPC of digested fractions was profiled on a column (10 × 300 mm) of Superdex Peptide 10/300 GL (GE Healthcare, Chicago, IL, USA). Elution was performed with 0.2 M NH_4_HCO_3_ at a flow rate 0.4 mL/min, and was monitored with a refractive index detector.

### 2.4. Phospholipids Hydrolysis

Phospholipids hydrolysis of resistant faction 2 was performed in 25 mM Tris-HCl pH 8.0 as 5–10 mg/mL, containing 1 mg/mL phospholipase A1 and 1 mg/mL phospholipase A2 at 37 °C for overnight. Then, phospholipases were removed by thermal precipitation. The supernatant was adjusted into pH 4.0 by HCl and then was extracted fatty acids portion by chloroform. After that, the supernatant was adjusted pH back into neutral for heparin lyase III digestion again. The fatty acids extraction was evaporated to dryness and treated with 1 N sodium hydroxide at 50 °C overnight to fully release the fatty acids.

### 2.5. NMR Spectroscopy

Samples (2.0 to 5.0 mg) were dissolved in 500 μL of D_2_O (99.9%) and lyophilized three times to substitute the exchangeable protons with deuterium, and then transferred to NMR microtubes after dissolving in 500 μL D_2_O. Resistant fraction 1 was analyzed by 1D ^1^H NMR and 2D NMR (^1^H-^13^C HSQC, ^1^H-^1^H COSY and TOCSY). Resistant fractions 1’, 2, and 2’ were analyzed by 1D ^1^H NMR. All NMR experiments were performed on Bruker 600 spectrometer (Bruker, Madison, WI, USA) with topspin 3.2 software at 298.15 K. 1D ^1^H NMR experiments were performed with 64 scans and 2 s acquisition time. 2D ^1^H-^13^C HSQC experiment was performed with 16 scans and 250 ms acquisition time. 2D ^1^H-^1^H COSY experiment was performed with 16 scans and 400 ms acquisition time. 2D ^1^H-^1^H TOCSY experiment was performed with 16 scans and 350 ms acquisition time.

### 2.6. Monosaccharide Analysis

The monosaccharide composition of resistant fraction 2’ was determined by the 1-phenyl-3-methyl-5-pyrazolone (PMP) derivatization followed by HPLC while referring the similar publication [17]. Briefly, resistant fraction 2’ and a heparosan tetrasccharide (ΔUA-GlcNAc-GlcA- GlcNAc) were hydrolyzed with 2 M trifluoroacetic acid (TFA) at 110 °C for 8 h. The hydrolyzate was derived in methanol solution of PMP at 70 °C for 30 min. The labeled carbohydrates were analyzed by HPLC with a mixture of 0.1 M KH_2_PO_4_ and CH_3_CN (83:17, *v*/*v*) as mobile phase. d-glucose, d-glucosamine, l-rhamnose, d-xylose, l-arabinose, d-mannose, l-fucose, d-galactose, and d-galacturonic acid were derived and used as standards.

### 2.7. Mass Spectroscopy

Glycan samples were dissolved in HPLC grade water as 0.2–0.5 μg/μL, and each sample (5 μL) was run in direct infusion mode by the standard ESI source of LTQ-Orbitrap XL FTMS (Thermo Fisher Scientific, San-Jose, CA, USA). LC parameters: Agilent Poroshell 120 ECC18 column (2.7 μm, 3.0 × 50 mm), mobile phase A was 5-mM ammonium acetate prepared with HPLC grade water, and mobile phase B was 5-mM ammonium acetate prepared in 98% HPLC grade acetonitrile with 2% of HPLC grade water. The flow was used 50% A and 50% B at a rate of 250 μL/min. The source parameters for FTMS were in the negative-ion mode, a spray voltage of 4.2 kV, a capillary voltage of −40 V, a tube lens voltage of −50 V, a capillary temperature of 275 °C, a sheath flow rate of 30 L/min, and an auxiliary gas flow rate of 6 L/min. All FT mass spectra were acquired at a resolution 60,000 with 200–2000 Da mass range. Fatty acids sample was dissolved in HPLC acetonitrile and used 100% B as flow; all other conditions were the same.

## 3. Results and Discussions

**Scheme 1:**
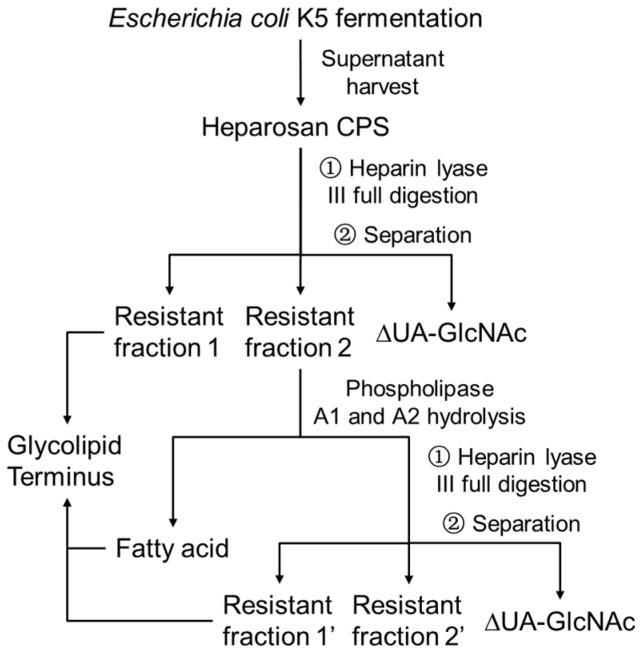
Flow chart of the *Escherichia coli* K5 heparosan capsular polysaccharide (CPS) processing and the resulting fractions for glycolipid terminus analysis.

**Figure 1:**
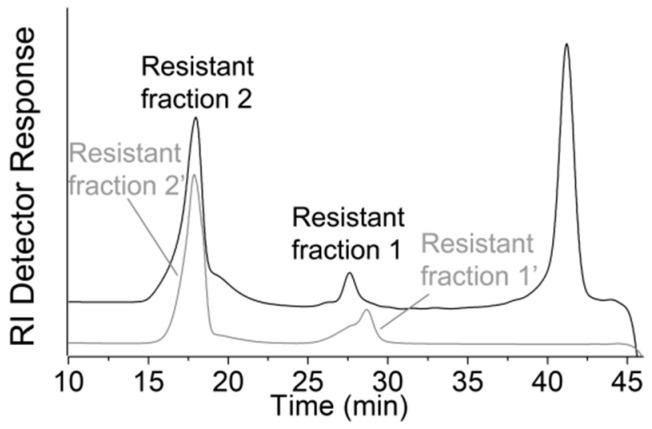
Black line: high-performance gel permeation chromatography (HPGPC) profiles of fully digested heparosan by heparin lyase III. Most of the disaccharides and digestion buffer salts have been removed by 1 kDa molecular weight cut-off (MWCO) spin column. Gray line: HPGPC profiles of heparin lyase III fully digested resistant fraction 2 after treatment with phospholipase A1 and A2. All of the disaccharides and digestion buffer salt have been removed by 1 kDa MWCO spin column.

A flow chart of the processing and the resulting fractions is shown in Scheme 1. *Escherichia coli* K5 was fermented on glucose, and heparosan CPS of molecular weight ~100 kDa was recovered from the supernatant and desalted [15]. Complete digestion of this heparosan with heparin lyase III afforded primarily the disaccharide ΔUA-GlcNAc (where ΔUA is 4-deoxy-*α*-l-*threo*-hex-4- enopyranosyluronic acid) along with two resistant fractions of higher molecular weight (Figure 1).

**Figure 2:**
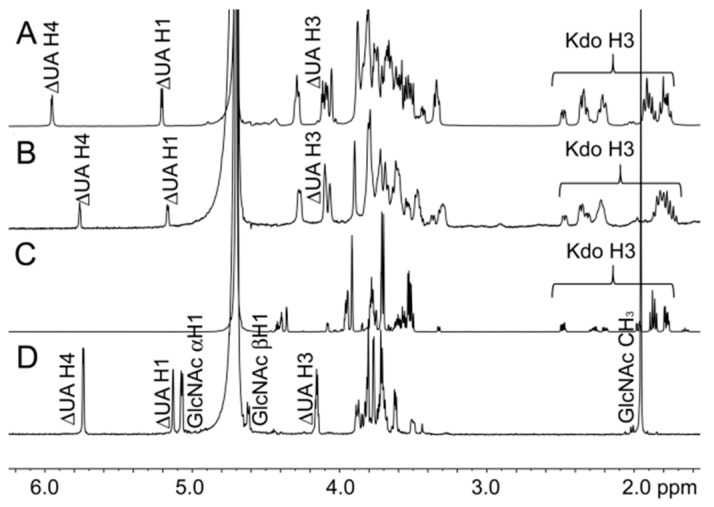
^1^H NMR spectrum of (**A**) resistant fraction 1, (**B**) resistant fraction 1’, (**C**) 3-deoxy-d-*manno*-oct-2-ulosonic acid (Kdo) standard, and (**D**) heparosan disaccharide (ΔUA-GlcNAc) standard. The ΔUA H4 of resistant fraction 1 is impacted by residual digestion buffer calcium ions exhibiting a slightly different chemical shift from that of resistant fraction 1’ and ΔUA-GlcNAc standard.

**Figure 3:**
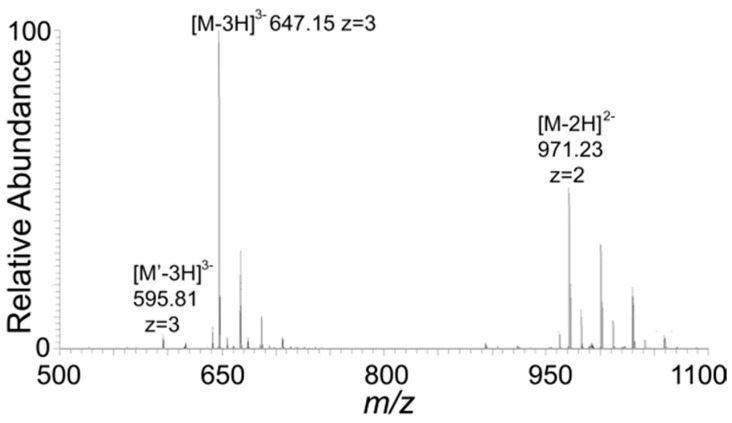
MS spectrum of resistant fraction 1. The spectrum matches that of a major component ΔUA-Kdo_7_-PG (M) and a very minor component ΔUA-Kdo_7_-glycerol (M’).

Based on the related publication [7], using Kdo standard (Figure 2C) and ΔUA-GlcNAc standard (Figure 2D), it is clear that resistant fraction 1 (Figure 2A) contains a single ΔUA residue at its non-reducing end and several Kdo residues. Furthermore, the mass spectrum of resistant fraction 1 (Figure 3) suggested it corresponded to the phosphatidylglycerol (glycerol-phosphate-glycerol, PG) reducing end of the CPS, which anchors it to the outer cell membrane [7]. Prominent ions at *m*/*z* 647.15 [M-3H]^3−^ and *m*/*z* 971.23 [M-2H]^2−^ match ΔUA-Kdo_7_-glycerol-phosphate-glycerol. All the other observed ions matched this structure when ammonium adduction from the buffer salt and dehydration was considered. A minor ion at *m*/*z* 595.81[M’-3H]^3−^ was assigned to ΔUA-Kdo_7_-glycerol, a fragment of ΔUA-Kdo_7_-PG.

**Figure 4:**
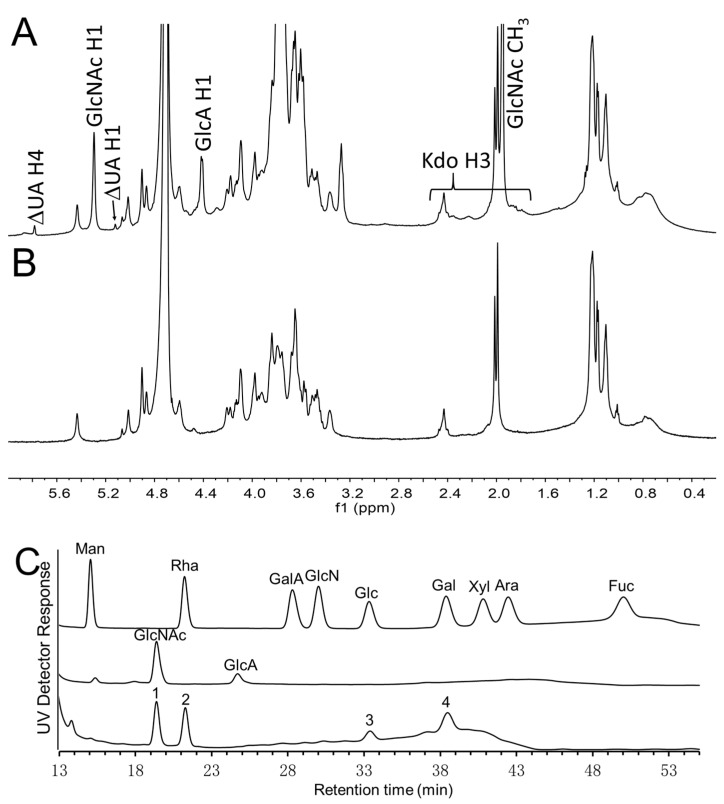
^1^H NMR spectrum of (**A**) resistant fraction 2 and (**B**) resistant fraction 2’. (**C**) Monosaccharide analysis of resistant fraction 2’. From top to bottom: nine kinds of monosaccharide standard, monosaccharide analysis of a heparosan tetrasccharide (ΔUA-GlcNAc-GlcA-GlcNAc), and monosaccharide analysis of resistant fraction 2’.

**Figure 5:**
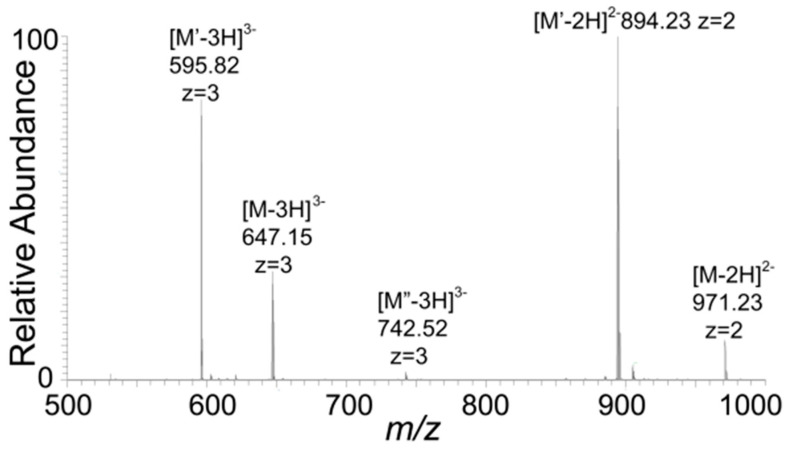
MS spectrum of resistant fraction 1’. The spectrum matches that of a major component ΔUA-Kdo_7_-glycerol (M’), a minor component ∆UA-Kdo_7_-PG (M), and a minor component ∆UA-Kdo_9_-glycerol (M’’).

The analysis result of resistant fraction 1 surprisingly suggests that the reducing terminus of *Escherichia coli* K5 heparosan CPS was different from that recently proposed by Willis and coworkers [7]. Instead of Kdo_7_, the Willis and coworkers study showed an even number of Kdo residues with the predominant fraction corresponding to Kdo_6_ and a minor fraction to Kdo_8_. Moreover, Willis and coworkers [7] showed the last Kdo residue to be glycosidically linked to GlcNAc not GlcA as suggested by our study. Based on these observed differences, we explored the lipid component by searching for a fragment containing the fatty acids (Scheme 1). ^1^H NMR spectrum of the resistant fraction 2 (Figure 4A) showed signals associated with the ΔUA, GlcNAc, GlcA, and Kdo residues and lipid. The presence of the GlcNAc and GlcA residues suggest that heparin lyase III was unable to fully depolymerize the lipidated heparosan as it did the heparosan linked to Kdo_7_-glycerol-phosphate-glycerol. We used phospholipase A1 and A2 to test this hypothesis by fully hydrolyzing the fatty acids on the terminal glycerol of resistant fraction 2, after which the sample was again exhaustively treated with heparin lyase III (Scheme 1). The result was the formation of a new resistant fraction 1’, with a slightly lower molecular weight, eluting slightly behind resistant fraction 1 (Figure 1). The ^1^H NMR spectrum of resistant fraction 1’ was nearly identical to the ^1^H NMR of resistant fraction 1 (Figure 2). MS analysis of resistant fraction 1’ (Figure 5) showed ions at *m*/*z* 647.15 [M-3H]^3−^ and *m*/*z* 971.23 [M-2H]^2−^, again corresponding to a structure as ΔUA-Kdo_7_-PG. The more intense signals at *m*/*z* 595.82 [M’-3H]^3−^ and *m*/*z* 894.23 [M’-2H]^2−^, corresponding to ΔUA-Kdo_7_-glycerol, suggest that the reducing terminal ‘’phospho-glycerol’’ was probably partially hydrolyzed on treatment with the phospholipases. This conversion of the PG moiety to the glycerol moiety explains the slight reduction in the molecular weight of resistant faction 1’ compared to resistant faction 1. An additional minor ion was observed at *m*/*z* 742.52 [M”-3H]^3−^, corresponding to ΔUA-Kdo_9_-glycerol.

The ^1^H NMR spectrum of the resistant fraction 2’ (Figure 4B), resulting from phospholipase A1 and A2 and heparin lyase III treatment of resistant fraction 2, showed no signals corresponding to ΔUA or Kdo H3 protons, demonstrating that all the Kdo chains had been released. The monosaccharide composition (Figure 4C) indicates that resistant fraction 2’ is mainly composed of GlcNAc (peak 1), rhamnose (peak 2), glucose (peak 3), and galactose (peak 4). We suggest that the resistant fraction 2’ comes from another cell membrane component, since it is mainly composed of fatty acids and a complex glycan. We next analyzed the free fatty acids that had been released, and they were almost completely comprised of C16:0 and C18:0 (Figure S2). We were unable to observe C18:1, described by Willis and coworkers [7].

Willis and coworkers [7] only collected and analyzed the Kdo chains with fatty acids, and the fatty acids preventing the full digestion and retaining several disaccharide (GlcA-GlcNAc) units. In the present report, that the Kdo chain without fatty acids was fully digested as resistant fraction 1 and the Kdo chain with fatty acids could not be fully digested as resistant fraction 2. The ^1^H NMR of resistant fraction 2 (Figure 4A) demonstrates that this is the case. After hydrolyzing the fatty acids and digesting again with heparin lyase III, resistant fraction 1’ was obtained with full digestion and exhibited the same Kdo chain structure as fraction 1. Our study provides a new and comprehensive view for analyzing the glycolipid terminus of *Escherichia coli* K5 CPS. However, there is no fundamental discrepancy between their and our analysis of the Kdo chain.

Thus, we carefully checked the liquid chromatography–mass spectrometry (LC-MS) data of Willis and coworkers [7] that suggests the inconsistency in the number of Kdo residues results from the first few repeat residues of the CPS connected to the Kdo repeat. The enzyme they used for depolymerizing CPS should have produced ΔUA-containing products based on the cited literature [18]. Thus, all of the terminal glycolipids they obtained would have a non-reducing end ΔUA instead of GlcA. Based on information only from LC-MS spectra, it is unclear whether the final Kdo is glycosidically linked to GlcA or GlcNAc. There were also problems in matching the expected *m*/*z* with the observed *m*/*z* in their low-resolution MS spectra. We believe that these factors led to an error in the structure of the terminal glycolipids obtained from *Escherichia coli* K5. Based on the first few residues from non-reducing end to reducing end being ΔUA-(GlcNAc-GlcA)_n_ (*n* = 1–3), the observed ions could be reassigned as shown in Table 1. All those Kdo chains observed would be comprised by 7 (major) or 9 (minor) Kdo residues and linked to C16:0 or C18:0 (rather than C18:1) fatty acids at the terminal glycerol, completely consistent with our proposed structure. During the production of bioengineered heparan sulfate or heparin from the *Escherichia coli* K5 heparosan precursor [9], the lipid terminus would be lost under the alkaline conditions used in partial de-*N*-acetylation of heparosan. By understanding the correct structure of the glycolipid at the reducing terminus of heparosan, we now can better understand how additional treatment, such as acidic or oxidative processing, can result in the removal of Kdo. Furthermore, knowledge of the correct terminal structure facilitates the monitoring of its removal by LC and LC-MS. Such analysis is required to demonstrate complete removal and show only a harmless level of residual glycolipid in heparan sulfate or heparin products.

It has been shown that two conserved *β*-Kdo transferases from the ATP-binding cassette (ABC) transporter-dependent system, KpsS and KpsC, are responsible for adding the Kdo residues on the (lyso-)PG acceptor located at the cytoplasm-membrane interface in some *Escherichia coli* and *N. meningitides* serotypes, including *Escherichia coli* K5 [19,20,21]. Biosynthesis is initiated by KpsS, which transfers a single Kdo residue to (lyso-)PG. Next, the KpsS product is extended by KpsC forming the *β*-Kdo linker. Full-length KpsC contains the N- and C-terminal domains catalyzing formation of *β*-2,4 and *β*-2,7 linkages, respectively. In vitro, the one-by-one addition of alternating *β*-2,4 and *β*-2,7 linked Kdo disaccharides to an acceptor was achieved by KpsC. Thus, Willis and coworkers have clearly found that CPS biosynthesis of Gram-negative pathogens containing KpsS and KpsC would produce odd numbers of Kdo residues. The subsequent work from their groups regarding the biosynthesis support our conclusion. However, limited by the error in their analysis of Kdo residues in *Escherichia coli* K5 [7], they conclude that these CPSs contain only odd or even numbers of Kdo residues, depending on the serotype. Now, our conclusion has clarified this ambiguity confirming that the KpsS and KpsC, a combination *β*-Kdo transferase from the ABC transporter-dependent system, catalyzes the formation of Kdo chains having odd numbers of Kdo residues.

We next confirmed the linkages of Kdo residues in ΔUA-Kdo_7_-glycerol-phosphate-glycerol using 2D NMR. Signals from the ΔUA residue were distinct from those of the Kdo residues (Figure S3). The H3 protons of the Kdo residues included the axial and equatorial protons, which were easily identified using ^1^H-^1^H correlation spectroscopy (COSY) (Figure S3A). Similarly, the H4 protons were identified based on their connection to the H3 protons using COSY. ^1^H-^1^H total correlation spectroscopy (TOCSY) (Figure S3B) was used to identify the H5 protons connected to the H3 and H4 protons. Due to spectral overlap, we were unable to identify the further protons. ^1^H-^13^C heteronuclear single quantum coherence spectroscopy (HSQC) (Figure S3C) was used to identify the corresponding carbon signals (Table 2).

We could divide the signals into two groups corresponding to *β*-2,4 linked Kdo residues and *β*-2,7-linked Kdo residues based on their H4 and C4 signals [21]. There were distinctive signals in Kdo residues at different positions but with the same linkage. Most of H3 signals showed a difference of >0.5 ppm for axial and equatorial orientations confirming the *β*-linkages of these residues [22]. The only group of H3 signals that showed a difference of 0.3 ppm, for the axial and equatorial orientations, was the reducing end Kdo *β*-2,4-linked to glycerol but its other protons and carbons signals remained unchanged. Thus, based on the working mechanism the KpsS and KpsC [20,21], our structural analysis of *Escherichia coli* K5 CPS confirmed its Kdo residues were connected through alternating *β*-2,4 and *β*-2,7 linkages.

## 4. Conclusions

Based on this study, we now provide a corrected structure for the linkage region of heparosan that anchors it to the outer membrane of *Escherichia coli* K5. This structure of *Escherichia coli* K5 heparosan CPS is GlcNAc *α*-1,[4GlcA *β*-1,4GlcNAc *α*-1,]_n_4GlcA *β*-1,[4Kdo *β*-2,7Kdo *β*-2,]_0 or 1_4Kdo *β*-2,7Kdo *β*-2,4Kdo *β*-2,7Kdo *β*-2,4Kdo *β*-2,7Kdo *β*-2,4Kdo *β*-PG-I (C16:0 or C18:0) (where n is ~250 for a CPS of 100 kDa).

## Figures and Tables

**Table 1:** Reassigning of ions in the LC-MS spectra for the terminal glycolipids of *Escherichia coli* K5 from the publication of Willis and coworkers [7].

Observed *m*/*z* ^a^	Species ^a^	Reassigned Species (Monoisotopic *m*/*z*)
853.2^3−^	l-PG(C16:0)-Kdo_6_-(GlcNAc-GlcA)_2_	l-PG(C16:0)-Kdo_7_-GlcA-GlcNAc-ΔUA (852.93^3−^)
862.1^3−^	l-PG(C18:1)-Kdo_6_-(GlcNAc-GlcA)_2_	l-PG(C18:0)-Kdo_7_-GlcA-GlcNAc-ΔUA (862.28^3−^)
979.6^3−^	l-PG(C16:0)-Kdo_6_-(GlcNAc-GlcA)_3_	l-PG(C16:0)-Kdo_7_-(GlcA-GlcNAc)_2_-ΔUA (979.30^3−^)
988.9^3−^	l-PG(C18:1)-Kdo_6_-(GlcNAc-GlcA)_3_	l-PG(C18:0)-Kdo_7_-(GlcA-GlcNAc)_2_-ΔUA (988.65^3−^)
829.3^4−^/1106.0^3−^	l-PG(C16:0)-Kdo_6_-(GlcNAc-GlcA)_4_	l-PG(C16:0)-Kdo_7_-(GlcA-GlcNAc)_3_-ΔUA (829.00^4−^/1105.67^3−^)
1115.2^3−^	l-PG(C18:1)-Kdo_6_-(GlcNAc-GlcA)_4_	l-PG(C18:0)-Kdo_7_-(GlcA-GlcNAc)_3_-ΔUA (1115.02^3−^)
1000.2^3−^	l-PG(C16:0)-Kdo_8_-(GlcNAc-GlcA)_2_	l-PG(C16:0)-Kdo_9_-GlcA-GlcNAc-ΔUA (999.64^3−^)
1126.7^3−^	l-PG(C16:0)-Kdo_8_-(GlcNAc-GlcA)_3_	l-PG(C16:0)-Kdo_9_-(GlcA-GlcNAc)_2_-ΔUA (1126.01^3−^)
1135.7^3−^	l-PG(C18:1)-Kdo_8_-(GlcNAc-GlcA)_3_	l-PG(C18:0)-Kdo_9_-(GlcA-GlcNAc)_2_-ΔUA (1135.35^3−^)
939.6^4−^/1252.9^3−^	l-PG(C16:0)-Kdo_8_-(GlcNAc-GlcA)_4_	l-PG(C16:0)-Kdo_9_-(GlcA-GlcNAc)_3_-ΔUA (939.03^4−^/1252.38^3−^)

^a^ Data are cited from the publication of Willis and coworkers [7]; l-PG indicates lyso-phosphatidylglycerol.

**Table 2:** Chemical shifts (ppm) of the Kdo residues from *Escherichia coli* K5 CPS.

Kdo Residues	H3 ax. (C3)	H3 eq. (C3)	H4 (C4)	H5 (C5)
→4)-*β*-Kdo*p*-(→2	1.89 (32.9)	2.51 (32.9)	3.87 (71.9)	4.11 (64.9)
	1.93 (32.3)	2.23 (32.3)	3.84 (71.9)	4.09 (65.3)
→7)-*β*-Kdo*p*-(→2	1.80 (34.1)	2.36 (34.1)	3.64 (67.2)	3.90 (64.9)
	1.82 (34.9)	2.38 (34.9)	3.66 (67.2)	3.91 (64.9)

## Supplementary Materials

The following are available online at https://www.mdpi.com/2218-273X/10/11/1516/s1, Figure S1: High performance gel permeation chromatography (HPGPC) profiles of partially digested heparosan by heparin lyase III, Figure S2: Mass spectrum of fatty acids hydrolyzed from resistant fraction 2 by phospholipase A1 and A2, Figure S3: (A) COSY NMR spectrum of the resistant fraction 1. (B) TOCSY NMR spectrum of the resistant fraction 1. (C) HSQC NMR spectrum of the resistant fraction 1.
