# Supplementary material for: A Revised Structure for the Glycolipid Terminus of Escherichia coli K5 Heparosan Capsular Polysaccharide
Source: Biomolecules. 2020 Nov 6;10(11):1516. doi: 10.3390/biom10111516 (PMC7694667; doi:10.3390/biom10111516)
Supplement: Supplementary file 1 [file biomolecules-10-01516-s001.pdf]

A Revised Structure for the Glycolipid Terminus of *E. coli* K5 Heparosan Capsular Polysaccharide

Lufeng Yan [1], Li Fu [2], Ke Xia [2], Shiguo Chen [1], Fuming Zhang [2], Jonathan S. Dordick [2] and Robert J. Linhardt [2,3,*]

[1] College of Biosystems Engineering and Food Science, National-Local Joint Engineering Laboratory of Intelligent Food Technology and Equipment, Zhejiang University, Hangzhou 310058, China;

[2] Department of Chemical and Biological Engineering, Center for Biotechnology and Interdisciplinary Studies, Rensselaer Polytechnic Institute, Troy NY, 12180, United States;

[3] Department of Chemistry and Chemical Biology, Center for Biotechnology and Interdisciplinary Studies, Rensselaer Polytechnic Institute, Troy NY, 12180, United States;

* Correspondence: linhar@rpi.edu; Tel.: +1-518-276-3404

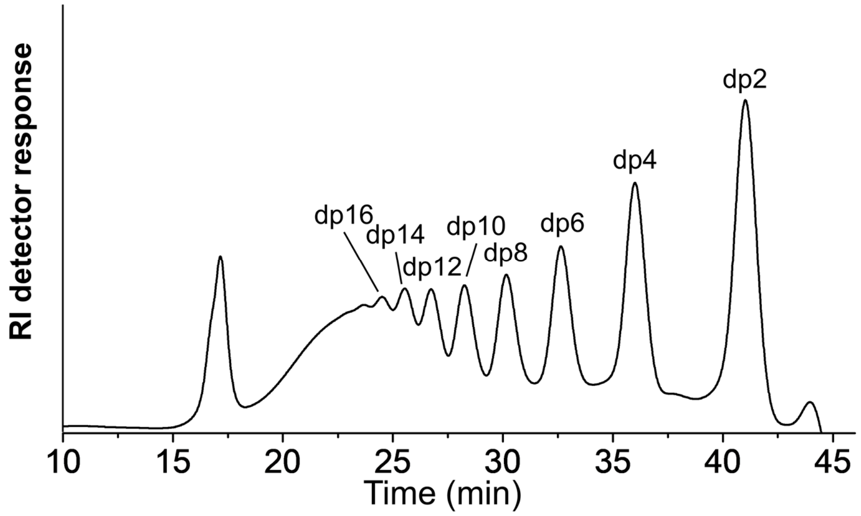

Figure S1. High performance gel permeation chromatography (HPGPC) profiles of partially digested heparosan by heparin lyase III.

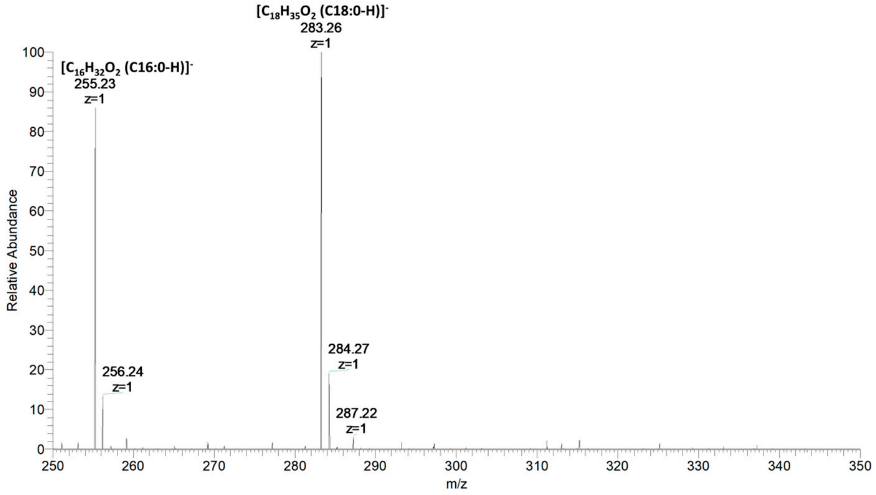

Figure S2. Mass spectrum of fatty acids hydrolyzed from resistant fraction 2 by phospholipase A1 and A2.

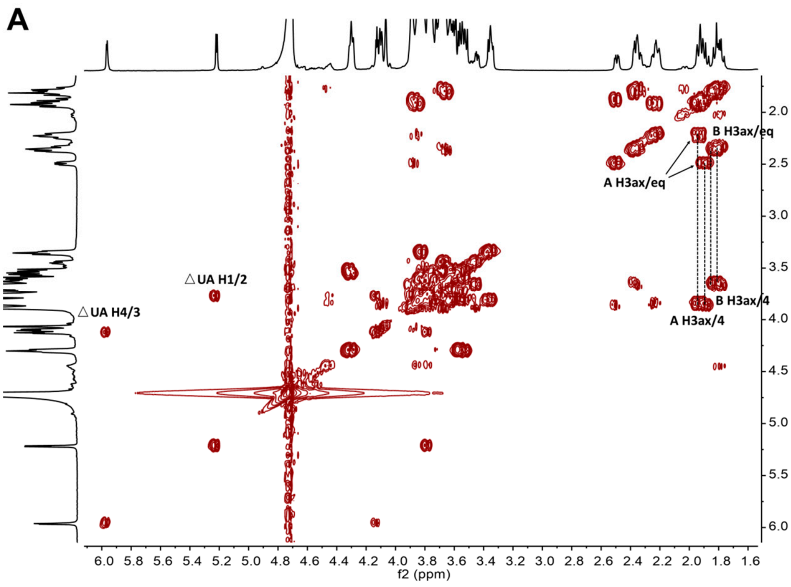

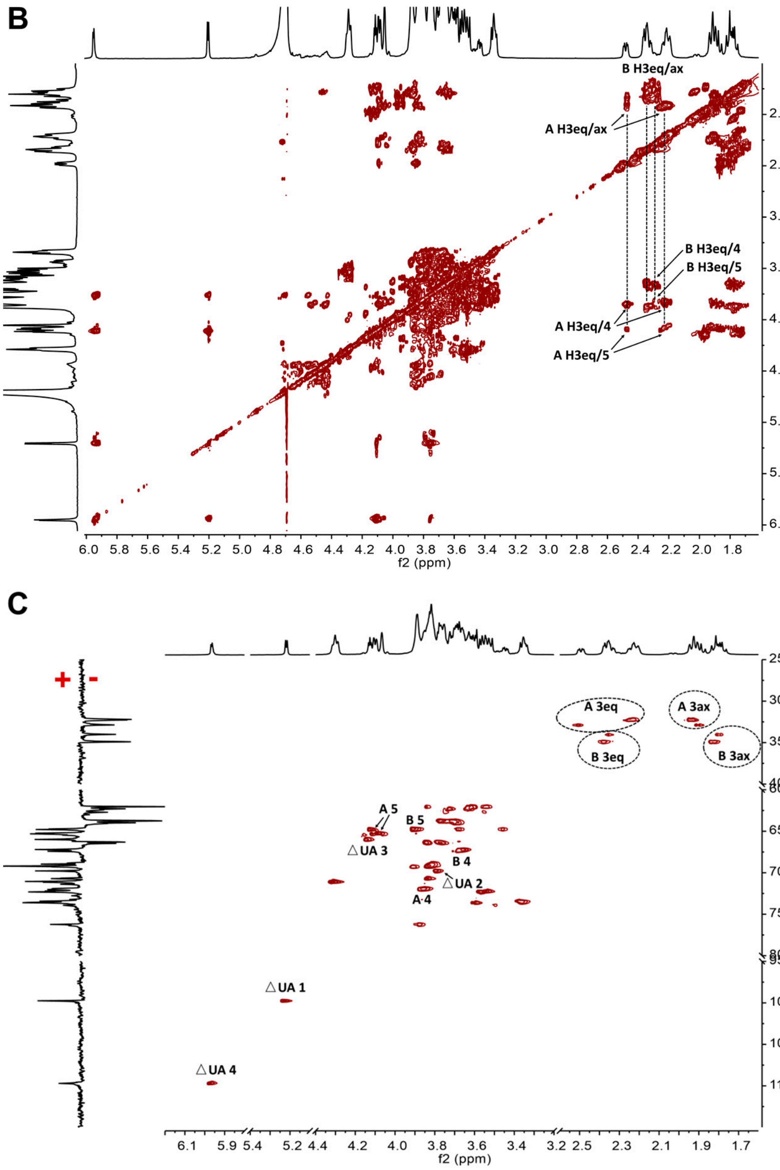

Figure S3. (A) COSY NMR spectrum of the resistant fraction 1. (B) TOCSY NMR spectrum of the resistant fraction 1. (C) HSQC NMR spectrum of the resistant fraction 1. Where, A is representative the residue of →4)-*β*-Kdo*p*-(→2, and B is representative the residue of →7)-*β*-Kdo*p*-(→2. The 'ax' is the abbreviation of 'axial', and the 'eq' is the abbreviation of 'equatorial'. In the ^{13}C spectrum of HSQC, the positive signals are from the carbons attached odd protons, and the negative signals are from the carbons attached even protons. In Figure S3C, to present a clearer version, some no signal areas (including 2.6-3.2 ppm, 4.4-5.1 ppm, 5.4-5.8 ppm in ^{1}H NMR and 40.0-60.0 ppm, 80.0-95.0 ppm in ^{13}C NMR) were cut off.
